# Who bears the brunt? Geographic, racial and ethnic disparities in mortality trends of inflammatory bowel disorders in the United States

**DOI:** 10.1017/cts.2025.10234

**Published:** 2026-01-06

**Authors:** Shamikha Cheema, Syed Ibad Hussain, Arun Kumar Maloth, Momina Khaliq, Muhammad Qasim, Muhammad Faique Hassan, Muhammad Shaheer Bin Faheem, Hasan Ijaz, Vicky Kumar

**Affiliations:** 1 King Edward Medical University, Lahore, Pakistan; 2 Jinnah Sindh Medical University, Karachi, Pakistan; 3 Kakatiya Medical College, Warangal, Telangana, India; 4 Shaheed Mohtarma Benazir Bhutto Medical College Lyari, Karachi, Pakistan; 5 Karachi Institute of Medical Sciences, Karachi, Pakistan; 6 George Washington University, Washington, DC, USA

**Keywords:** Inflammatory bowel diseases, Crohn’s disease, ulcerative colitis, geographical differences, CDC

## Abstract

**Background:**

Inflammatory bowel disease (IBD), encompassing ulcerative colitis (UC) and Crohn’s disease (CD), presents increasing global health burdens. Despite advancements in therapy, disparities in mortality trends across demographic and geographic lines persist in the United States.

**Objective:**

To analyze IBD-associated mortality trends in the U.S. from 2018 to 2023 using CDC WONDER data, highlighting demographic, regional, and sex-based disparities.

**Methods:**

A retrospective analysis of death certificate data from the CDC WONDER database was performed. Age-adjusted mortality rates (AAMRs) were calculated and stratified by sex, race/ethnicity, and region. Trends were evaluated via join-point regression, with the annual percentage change (APC) and average annual percentage change (AAPC) calculated to assess statistical significance.

**Results:**

A total of 25,153 IBD-related deaths were recorded. The AAMR increased from 8.269 (2018) to 10.761 (2023), with a notable increase until 2022 (APC: +8.91), followed by a decline in 2023 (APC: −7.55). Men presented higher AAMRs than women did (10.882 vs. 9.838). Non-Hispanic White individuals had the highest AAMR (11.401), whereas Non-Hispanic Black and Asian populations presented the steepest increases (APC: 10.49 and 13.45, respectively). Regionally, the Midwest had the highest AAMR (11.531), with Oregon demonstrating the highest state-level mortality.

**Conclusions:**

This study reveals increasing IBD mortality in the U.S., with significant sex, racial, and geographic disparities. These findings highlight systemic inequities in healthcare access, particularly in access to biologic therapy and specialty care. Targeted public health strategies are crucial for reducing disparities and enhancing outcomes in high-risk populations.

## Introduction

Inflammatory bowel disease (IBD) – Ulcerative colitis (UC) and Crohn’s disease (CD) – are long-term immunological illnesses characterized by the hallmark signs of gastrointestinal tract inflammation [1]. The highest yearly incidence of UC is 19.2/100,000 person-years in North America, 6.3/100,000 person-years in Asia and the Middle East, and 24.3/100,000 person-years in Europe. On the other hand, the greatest yearly incidence of CD was shown by North America at 20.2/100,000 person-years, Asia and the Middle East at 5.0/100,000 person-years, whereas Europe at 12.7/100,000 person-years. The maximum IBD incidence rates ever recorded are in Europe [2].

In the United States, the burden of inflammatory bowel disease (IBD) has shown persistent variation across time and population groups. Recent analyses indicate that although age-standardized mortality rates for IBD have declined over the past two decades, they remain substantial compared with other high-income countries [3]. Studies using nationwide datasets have highlighted geographic and sociodemographic disparities: higher hospitalization and mortality rates are consistently observed in rural areas, among individuals of lower socioeconomic status, and among certain racial and ethnic minorities [4]. Previous research also suggests that differences in healthcare access, insurance coverage, and treatment initiation contribute to these disparities in outcomes. Together, these findings underscore the ongoing need for targeted public health strategies to reduce inequities in IBD morbidity and mortality within the U.S.

According to research, IBD has been linked to early childhood otitis media, passive tobacco use, and exposure during pregnancy to age five [5]. An urban lifestyle is another risk factor responsible for the development of CD and UC [1]. The fact that more than 25% of IBD cases occur before the age of 18 among adolescents implies an unsettling rise in the disorder’s prevalence and severity [6]. There has been a notable global decline in the incidence of IBD especially the 50–69 age brackets, with rising rates of occurrence in men, particularly in the Asia–Pacific region [7]. On the other hand, there tend to be decreasing patterns among females [7]. The frequency and phenotypic manifestation of IBD may be altered by variables in the environment, as shown by geographic variations, population migration, and shifting patterns of disease occurrence [8].

A study was conducted in which 62,310 deaths from IBD were documented. Between 1999 and 2020, the CD age-adjusted mortality rate (AAMR) increased from 0.79 to 0.97 per 100,000 and then declined between 1999 and 2018 before rising again from 2018 onward. The reported AAMR was greater for women (0.81) than for men (0.77) [9]. However, emerging evidence indicates that rural communities experience disproportionately higher IBD-related mortality, likely due to limited access to gastroenterology specialists, delayed diagnosis, fewer advanced therapeutic options, and inadequate continuity of care [10]. Our research aims to fill this gap by focusing on the trends and disparities in UC and CD mortality from 2018 to 2023.

Examining the associations between IBD patient death rates and other epidemiologic characteristics, such as geographic location within the US, is essential to better understand the impact that IBD has on patients and the US healthcare system. Using data from the Centers for Disease Control and Prevention’s Wide-ranging Online Data for Epidemiologic Research (CDC WONDER) database, this study examines patterns to provide important information on regional trends in IBD mortality in the U.S. healthcare system in the U.S.

## Methods

The CDC WONDER database, a comprehensive online resource created for epidemiological studies, was utilized in this work. This program, which was created by the CDC, provides access to public health data at large [11]. We evaluated the Centers for Disease Control and Prevention Wide-ranging Online Data for Epidemiologic Research (CDC WONDER) database to identify fatalities associated with inflammatory bowel disease (IBD) that occurred in the United States.

Since only anonymized, deidentified and depersonalized data are included in the publicly available CDC WONDER database, this study is exempt from Institutional Review Board authorization as there is no risk to the involved subjects.

IBD was identified as underlying cause or contributing cause on death certificates of patients obtained from publicly available CDC WONDER multiple cause of death database. Any patients with IBD mentioned on their death certificates as either the underlying or contributing cause of death were included in our study. If IBD, as a comorbidity, significantly contributed to the death of the patient, it was considered as a contributing cause. The data was manually extracted from the website by 2 authors independently and verified by a third author. Prior investigations employing CDC WONDER to examine mortality trends in patients with inflammatory bowel disease have applied this type of approach [9,12]. The ICD codes used were K50.0, K50.1, K50.8, K50.9, K51.0, K51.1, K51.2, K51.3, K51.4, K51.5, K51.8, and K51.9, with no restrictions on age. Data were gathered on population size and deaths associated with IBD from 2018 to 2023. The retrieved geographical and regional group data consisted of age, Hispanics, race, census region, gender, place of death, and state. According to the definitions of the U.S. Census Bureau, the regional data were divided into four distinct groups: Northeast, Midwest, South, and West. The location of death was classified as unknown, outside of medical settings (home, nursing home/long-term care establishment, hospice facility), or within healthcare settings (outpatient/ER, inpatient). The races included American Indian, Asian, black or African, and white.

Age-adjusted mortality rates (AAMRs), and crude death rates associated with IBD were calculated by applying age-specific death rates for a population to the US 2000 Standard Population, to account for differences in age structure between various populations. This process involved calculating the death rate for each age group in the observed population, then multiplying those rates by the corresponding proportion of people in that age group in the standard population. The sum of these products provided the age-adjusted rate. Crude mortality rates were calculated by dividing the number of fatalities from IBD by the comparable population in the United States. The join-point regression program was used to determine trends in mortality over the investigation period. In particular, this software conducts a time series analysis by seeking statistically noteworthy variations in one-year mortality patterns via join-point regression modeling. The simplest model (a single linear trend) is the starting point for join-point modeling, which joins several linear segments at inflection points (join points) to split the data chronologically and show various instances when deviation occurs. The Monte Carlo permutation test was used to determine the annual percentage change (APC) in the AAMR for each line segment connecting join locations, along with the 95% CI. To illustrate the reported mortality trend across the entire research period, the weighted average of the APCs was also computed and presented as the average annual percentage change (AAPC) with 95% CIs. The 2-tailed *t* test was used to evaluate whether the value was significantly different from zero, indicating increases or decreases in the APC and AAPC. An asterisk (*) indicates statistically significant values, which were determined using a p value of ≤0.05.

## Results

Between the years 2018 and 2023, a total of 25,153 deaths related to ulcerative colitis (UC) and Crohn’s disease (CD) were recorded among the general population, encompassing individuals aged <1 to 85 years as well as those older than 85 years (Supplemental table 1). Information on the location of death was available for all 25,153 cases. Among the total UC and CD deaths, the most common place of death was at home, for 34.18% of cases. Nearly 33.92% occurred within medical facilities, including hospitals and inpatient care settings. Nursing homes and long-term care facilities were 14.91% of individuals. Hospices, providing end-of-life care, were the location for 9.00% of deaths. A smaller proportion of deaths occurred in emergency departments or outpatient settings, comprising 4.02% of the total. Additionally, 0.14% of patients were declared dead on arrival. Lastly, 3.82% of deaths occurred in locations that were either not documented or categorized under unnoted reasons, indicating a minor yet present gap in the granularity of reporting. (Supplemental table 2).

### Annual trends for UC and CD related AAMR

**Figure 1.**
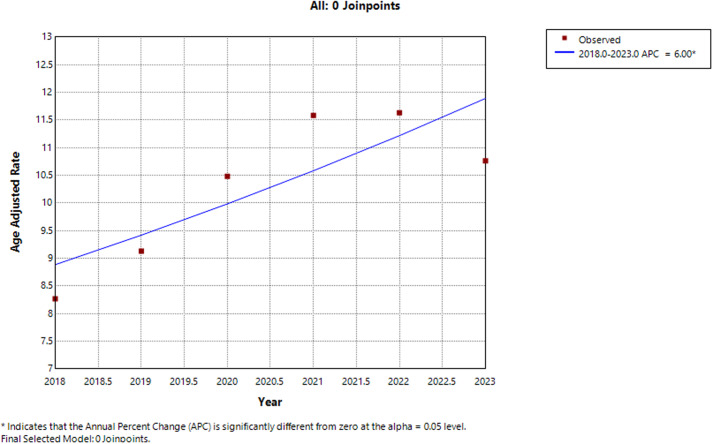
Join-point analysis of IBD-related mortality in the US from 2018–2023.

The AAMR for UC and CD related deaths in the general population was 8.269 per 1000,000 individuals in 2018 and increased to 10.761 per 1000,000 in 2023. Over the six-year period, AAMR demonstrated an overall upward trend. From 2018 to 2022, the AAMR increased from 8.269 in 2018 to 11.632 in 2022, noting notable increase in AAMR. However, a decline was seen from 11.632 in 2022 to 10.761 in 2023, showing a slight decrease in AAMR in general population. Overall, an increase in AAMR with an annual percentage change (APC) of 6.00* (95% CI: 0.05–12.30; *p* = 0.0488) was noticed, marking a statistically significant rise in mortality from 2018 to 2023 (Figure 1, Supplemental table 3).

### UC and CD related AAMR stratified by sex

**Figure 2.**
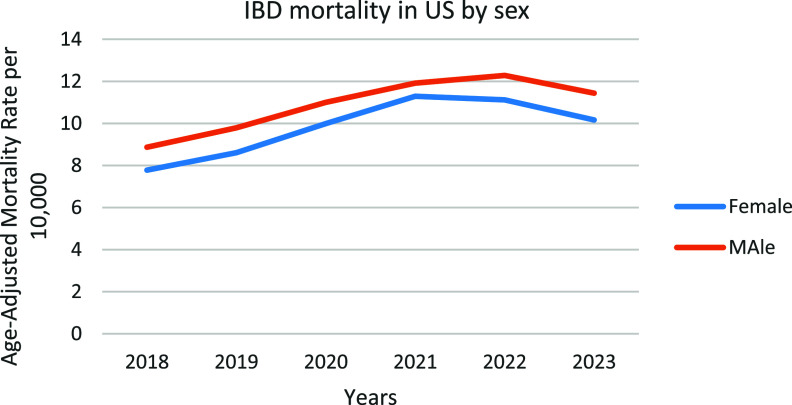
Trends of IBD-related mortality by sex in the US from 2018–2023.

Men in general had consistently higher AAMRs than women throughout the study period (overall AAMR men: 10.909; 95% CI: 10.708–11.109; women: 9.825; 95% CI: 9.655–9.996). In 2018, the AAMR for men was 8.868 which significantly increased to 11.436 in 2023 (APC: 5.69*; 95% CI: 0.74–10.89; *p* = 0.033). Similarly, the AAMR for women in 2018 was 7.778 which steadily increased but not significantly to 10.165 in 2023 (APC: 6.20; 95% CI: −0.689–13.588; *p* = 0.067) (Figure 2, Supplemental table 4).

### UC and CD related AAMR stratified by race/ethnicity

**Figure 3.**
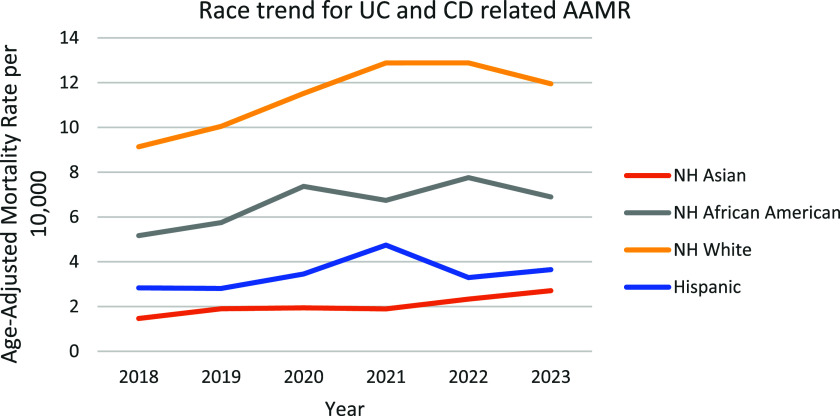
Trends and disparities in IBD-related mortality stratified by race in the U.S. From 2018–2023.

When stratified by race/ethnicity, AAMRs were highest among NH white patients followed by NH Black or African American, NH American Indian or Alaska Native, Hispanic or Latino, and NH Asian populations. The AAMR for NH white, increased from 9.135 in 2018 (95% CI: 8.801–9.469) to 12.88 in 2021 (95% CI: 12.484–13.277) followed by a constant AAMR of 12.88 till 2022 (95% CI: 12.489–13.268), with a decline to 11.948 in 2023 (95% CI: 11.575–12.321), with an overall change in the mortality rate over the course (APC: 6.182*: 95% CI: 0.204–12.516; *p* = 0.045) marking a significant rise in AAMR. The AAMR for NH Black or African American increased from 5.167 in 2018 (95% CI: 4.458–5.875) to 7.368 in 2020 (95% CI: 6.547–8.19), followed by a decrease to 6.742 in 2021 (95% CI: 5.944–7.54). The rate then increased to 7.758 in 2022 (95% CI: 6.919–8.597) before declining to 6.897 in 2023 (95% CI: 6.116–7.678). Overall, from 2018 to 2023, the AAMR showed a non-significant upward trend (APC: 6.12, 95% CI: −1.67–14.53; *p* = 0.097). The AAMR for NH American Indians decreased from 6.263 in 2019 (95% CI: 3.771–9.781) to 5.386 in 2021 (95% CI: 3.29–8.319), followed by an increase to 5.52 in 2022 (95% CI: 3.46–8.358). Overall, from 2019 to 2022, the AAMR declined (APC: −4.76, 95% CI: −11.887–2.951). The AAMR for NH Asians increased from 1.467 in 2018 (95% CI: 0.967–2.134) to 1.94 in 2020 (95% CI: 1.386–2.641), declined slightly to 1.894 in 2021 (95% CI: 1.353–2.579) and then a continuous incline from 1.894 in 2021 (95% CI: 1.353–2.579) to 2.708 in 2023 (95% CI: 2.066–3.486). Overall, from 2018 to 2023, the AAMR had increased significantly from 1.467 to 2.708 (APC: 11.11, 95% CI: 5.42–17.11; *p* = 0.005). The AAMRs for Hispanics decreased from 2.831 in 2018 (95% CI: 2.286–3.376) to 2.808 in 2019 (95% CI: 2.288–3.328), then a rapid rise was seen to 4.744 in 2021 (95% CI: 4.07–5.418), declining to 3.291 in 2022 (95% CI: 2.751–3.832) and rose to 3.651 in 2023 (95% CI: 3.081–4.221). Overall, AAMR increased non-significantly from 2.831 in 2018 to 3.651 in 2023 (APC: 5.684; 95% CI: −7.812–21.154; *p* = 0.324). (Figure 3, Supplemental table 5).

### UC and CD-related AAMRs stratified by geographic region

**Figure 4.**
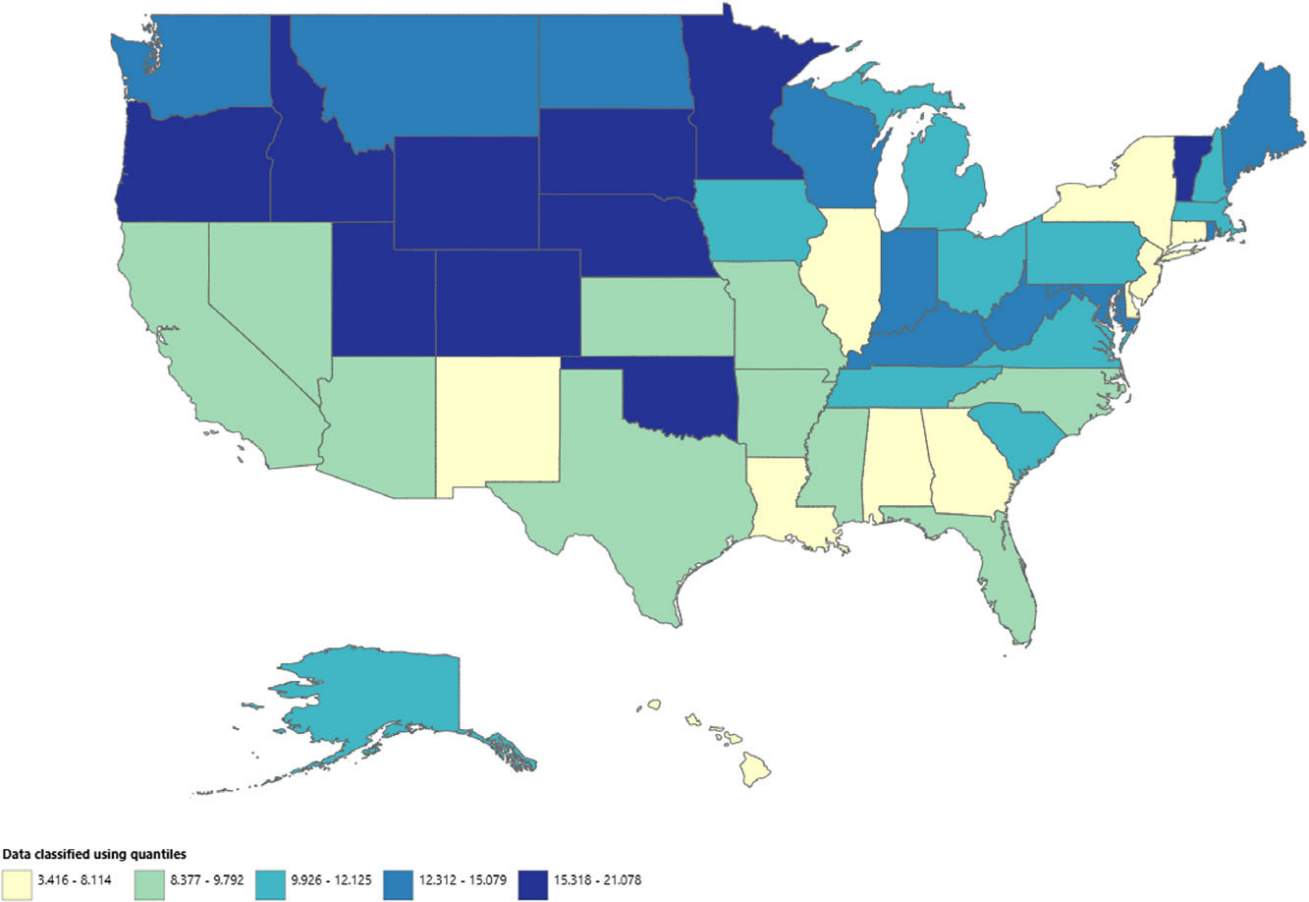
Overall ulcerative colitis and Crohn’s disease–related AAMRs stratified by states in the United States, 2018–2023.

**Figure 5.**
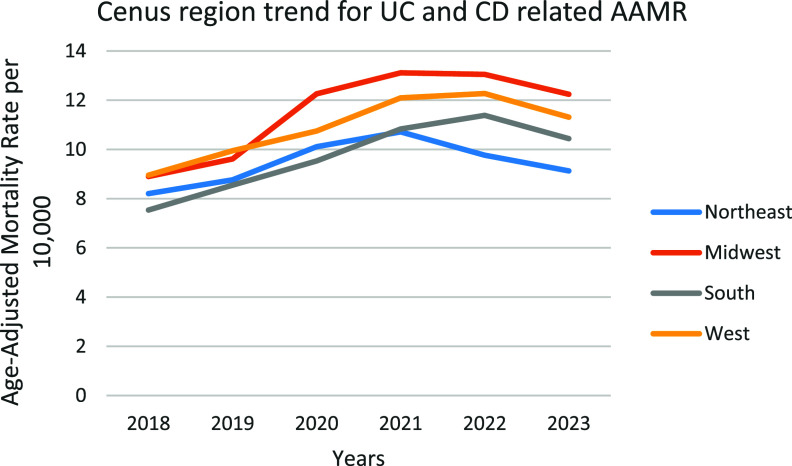
Trends and disparities in IBD-related mortality in the US from 2018–2023 stratified by census region.

A significant difference in AAMR was observed in different states, with the AAMRs ranging from 3.416 (95% CI: 2.417–4.689) in Hawaii to 27.237 (95% CI: 19.481–22.676) in Oregon. States that fell into the top 90th percentile were Oregon, Vermont, Rhode Island, Colorado, and Minnesota, which had approximately double the AAMRs compared with states that fell into the lower 10th percentile, namely, Hawaii, Florida, the District of Columbia, Arizona, and New Jersey (Figure 4, Supplemental table 6). In regions, over the course of the study period the highest mortality was observed in the Midwestern region (AAMR: 11.545, 95% CI: 11.246–11.844), with a non-significant rise in mortality (APC: 7.128; 95% CI: −0.380–15.202; *p* = 0.058). Western regions had the 2nd highest mortality rate (AAMR: 10.891; 95% CI: 10.612–11.17), with a significant increase in mortality (APC: 5.414*; 95% CI: 0.275–10.817; *p* = 0.043). Southern regions had the 3rd highest mortality rate (AAMR:9.742; 95% CI: 9.537–9.948), with a significant rise in mortality over the period (APC: 7.417*; 95% CI: 1.619–13.546; *p* = 0.023). Northeastern regions had the lowest mortality rate in all the regions (AAMR: 9.451; 95% CI: 9.16–9.741), with a non-significant rise in AAMR (APC: 2.416; 95% CI: −4.129–9.408; *p* = 0.372). (Figure 5, Supplemental table 7)

## Discussion

This analysis on the relationship between inflammatory bowel disease (UC and CD) in adults aged >1 to 85+ years and mortality through CDC Wonder demonstrates vital findings. Over the course of the period from 2018 to 2023, a total of 25,153 deaths were linked to UC and CD, with significantly higher mortality among males in comparison to females. In the US, the total number of IBD-related deaths, comprising UC and CD, has increased from approximately 2171 to 5910, an increase of roughly 172%, and presents with an increasing risk of mortality due to IBD [13]. The AAMR had increased from 2018 to 2023 in general, with variations in the AAMR and APC. These findings indicate that relying on broad mortality data may underestimate UC and CD’s impact on overall mortality rates, highlighting the importance of disease-specific mortality assessments in public health surveillance for better treatment and campaign programs directed at IBD.

In the U.S., cause of death information is determined by medical examiners based on clinical findings and circumstances at the time of death. Death certificates record underlying causes, immediate causes, and contributing causes of mortality, which might leave out IBD, leading to the omission of mortality due to IBD [14]. Inflammatory bowel disease (IBD) comprises UC and CD, which refer to chronic, inflammatory disorders of the gastrointestinal tract. IBD is driven by multiple factors. IBD results from a dysregulated mucosal immune response to intestinal microbes in genetically susceptible hosts [15], and environmental risk factors, including smoking, diet, antibiotic exposure, and sedentary lifestyle, contributed to IBD development [16]. Similar to other chronic diseases, IBD-related mortality risk increases with age, especially with comorbidities such as cardiovascular disease, diabetes, or malignancy [17,18]. Lifestyle-related factors influence the mortality rate, including smoking, obesity, diet, and medication adherence, which influence disease severity and progression [19].

The analysis indicated that IBD-related deaths occurred at home and, secondly, at medical facilities. Many of the deaths occurring at home between 2018 and 2023 imply that some of the years were during the COVID-19 pandemic, which would have caused fear amongst the patients of contracting COVID-19 within the medical facilities, as patients delayed their treatment programs [20]. The medical facilities were overwhelmed with the surge of COVID-19 patients, leading to fewer diagnostics and treatment being issued for IBD patients [21]. For instance, an article in 2020 discusses how the healthcare facilities shifted towards COVID-19 compared to emergency conditions, showing the impact of COVID-19 on patients with other conditions, including IBD [22]. High mortality in medical facilities implies that IBD patients developed hospital-acquired infections, which increases the mortality, since these conditions largely increase the risk of mortality [23]. Many IBD patients develop different diseases through the course of the disease, for instance, sepsis. This is because IBD patients arrive at the hospitals beforehand, which leads them to develop a new disease. Sepsis is one of the most common causes of death in IBD, according to a study in 2020 [24].

Furthermore, analysis revealed higher age-adjusted mortality rates for men than for women. This male predominance is consistent with Shah et al.’s broader meta-analysis of 42 studies, indicating a 21% greater risk of mortality for males [25]. The differences were driven by several compounding factors, with Kochar et al. reporting that men experience 38% greater rates of stricturing/penetrating complications than females [26]. Furthermore, CDC surveillance data suggest that women have a 52% advantage over men with respect to the development of biologic therapy [27]. Cohen-Mekelburg’s group estimated that 28% of excess male mortality is due to the delays in accessing care [28]. These trends were particularly pronounced for men under 50 years of age (7.2% change versus 5.1% for women), confirming Adler et al.’s cautionary guidance on the vulnerability of younger males [29]. Taken together, these findings emphasize Cross et al.’s call for male-informed, sex-specific interventions that improve early diagnosis through screening guidelines, improve treatment access through healthcare policies, and improve delays in help-seeking characteristics based on education programs targeted at adult male patients with IBD [30]. Males usually have narrower pelvises and more visceral fat compared to females, which may complicate pelvic surgeries like proctectomy, which therefore increases the complications arising after surgeries for males [25]. Higher smoking and alcohol use in men worsen wound healing, infections, and surgical recovery [19].

The mortality patterns revealed significant differences among the racial groups, as NH White individuals had the highest mortality, and NH Asian individuals had the lowest mortality. Most surprisingly, NH Black had a 10.49% yearly increase, which was double the annual increase reported by Damas et al. (5.2%) [31]. NH Asians had the greatest increase in deaths, thereby reversing the protective effect identified by Aniwan et al. in first-generation immigrants [32]. These findings also aligned with systemic disparities recorded in the literature, as Cohen-Mekelburg et al. reported that NH Black patients were 42% less likely to receive biologics for inflammatory bowel disease [28]. Emergent dietary adaptation may also have contributed to these findings, and previous research has indicated that transitioning populations from Western dietary habits increased the risk of hospitalization due to inflammatory bowel disease by 2.3-fold [33,34]. The data substantiates the urgent need for structural interventions to address the shortage of specialists; 57% of the rural counties had no gastroenterologists, insurance disparities, or culturally competent care models [29,35]. Racial disparity and racism amongst the NH black individuals led to an increase in the AAMR over the years. This is due to the fact that NH black individuals are provided with fewer healthcare facilities compared to other ethnicities within the US [36]. Whereas, NH Asians have lower prevalence of obesity, diabetes, hypertension, and smoking among themselves compared to other groups, as well as have better lifestyles than other ethnicity groups due to their standard of living and diet. For example, NH Asians in general have higher rates of health insurance compared to other races [37].

The analysis revealed significant geographical differences in IBD mortality, with the Midwest showing the largest burden. These marked differences reflect the multiple synergistic factors shown in the previous literature; notably, the shortage of specialists is particularly severe in rural areas, where the average number of gastroenterologists per 100,000 residents in high-mortality areas is only 1.2 [29], and the average diagnostic delay is staggering 14.2 months for states within the worst ranges [38]. Additionally, environmental factors play a crucial role, and the risk for IBD increased by 18% (RR: 1.18) for every 5 μg/m^3^ increase in PM2.5 exposure. Ananthakrishnan et al. provided strong support for urban versus rural systems of care, which contrasts poorly with the project-level cardiovascular outcomes published by the American Heart Association, and those findings were not as pronounced, as there was a poor distinction between urban versus rural rates of IBD mortality and proximity to emergency services [39]. However, if access is maintained in time-limited hospital-based specialties and urgent-based care, the potential to improve access to living that is living 7 years longer than the average across all states is outstanding. Concerning the issue for rural area residents is that there were closures of healthcare facilities within the US, as from 1990 to 2020, around 334 hospitals have been closed within the US, leading to patients not being taken care of properly care [40]. A study in the *American Journal of Public Health* highlights that rural local health departments operate with significantly less local tax revenue, placing them at a disadvantage for delivering essential health services for the patients within their areas. This causes a lack of required equipment for the treatment [41]. This warrants a special focus on creating telemedicine hubs retained in underserved areas, defining environmental health programs that target higher risk areas, and finally reviewing and documenting state-based policies that better target insurance coverage and specialists [30].

### Clinical implications

Our findings emphasize the urgent need for fair access to gastroenterology care, especially for racial minorities and populations in high-burden regions. Early diagnosis and timely initiation of biologic therapy could mitigate the observed disparities in IBD-related mortality. Public health interventions, including telemedicine expansion and culturally tailored care models, are critical to reducing inequities. Policymakers must prioritize resource allocation to underserved communities to improve survival outcomes in IBD patients, with allocations of resource to aged patients.

### Limitations

Our study is retrospective and based on CDC World death certificate data, which can introduce subjectivity or misclassification in reporting mortality related to IBD. Clinical data about disease duration, therapies, adherence, or specific patient condition data were lacking, making it difficult to explore the drivers of mortality fully. The COVID-19 pandemic (2020–2022) may have led to differential mortality metrics because of disruptions in healthcare access. While some limitations were highlighted, our study provides the most recent and thorough investigation of IBD-related mortality (2018–2023) with US population regarding ulcerative colitis and Crohn’s disease in these societal racial/ethnic groups and regions by increasing broader applicability.

## Conclusion

We observed noteworthy demographic and geographic differences in IBD-associated mortality, with overall, male, non-Hispanic Black, and Asian patients experiencing higher mortality. The overall mortality rate decreased in 2023; nevertheless, the overall rising trend in deaths, particularly with populations of concern that are marginalized, indicates the compelling need for specific interventions in the populations of concern. These results can be attributed to systemic care barriers, such as disparities in access to specialty care and biologic therapy among racial-ethnic minorities and indicate real differences in outcomes. To reduce health disparities, culturally responsive care models, enhanced surveillance, and continued policy advocacy will be central to achieving health equity for vulnerable IBD patients across the United States.

## Supporting information

10.1017/cts.2025.10234.sm001 Cheema et al. supplementary material Cheema et al. supplementary material

## Data Availability

Not Applicable.

## Supplementary material

The supplementary material for this article can be found at https://doi.org/10.1017/cts.2025.10234
