# Supplementary material for: Who bears the brunt? Geographic, racial and ethnic disparities in mortality trends of inflammatory bowel disorders in the United States
Source: J Clin Transl Sci. 2026 Jan 6;10(1):e16. doi: 10.1017/cts.2025.10234 (PMC12895456; doi:10.1017/cts.2025.10234)
Supplement: Cheema et al. supplementary material [file S2059866125102343sup001.docx]

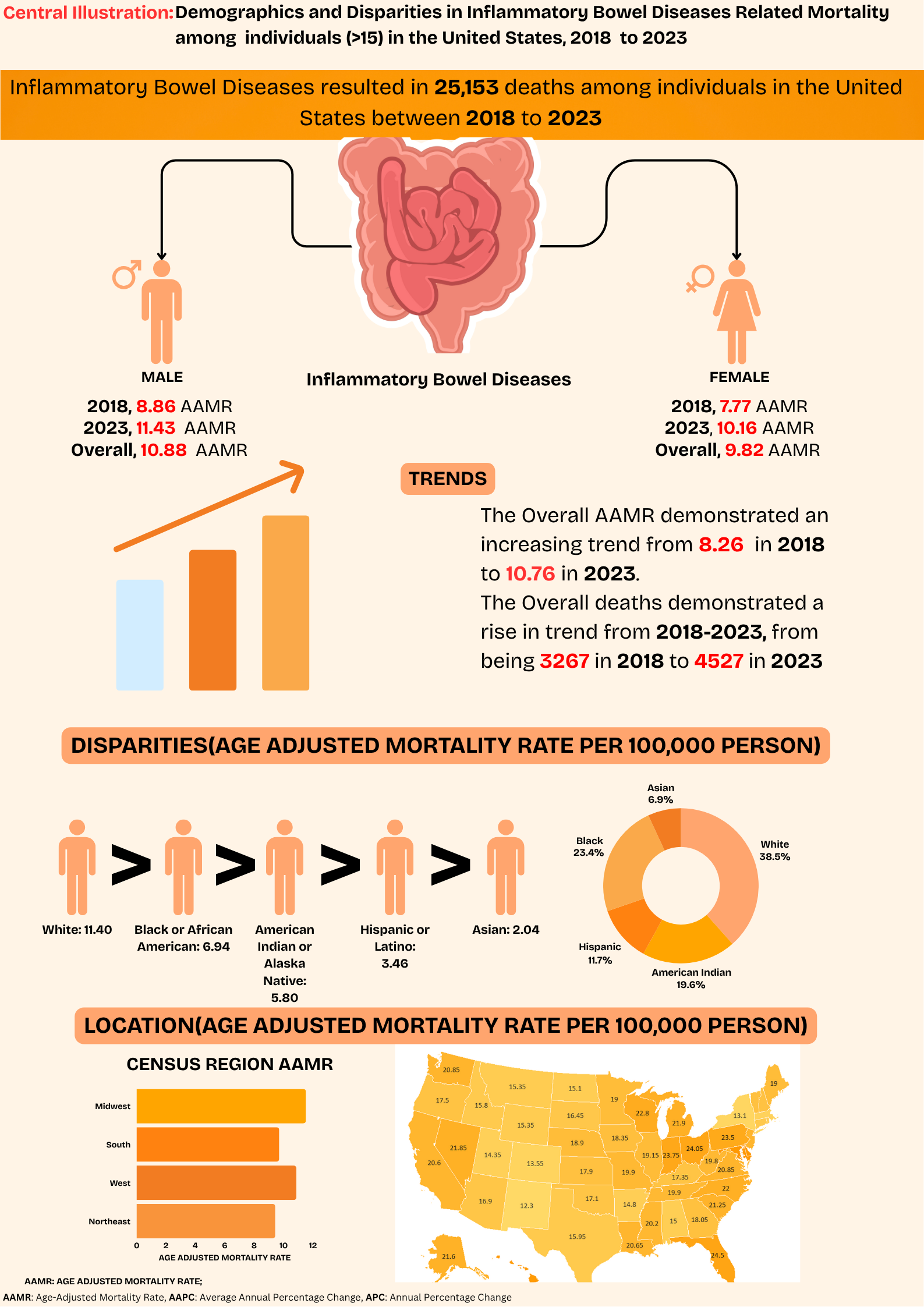


**Supplemental Table 1** UC and CD-related deaths, stratified by sex and race, in general population in the United States, 2018 to 2023

| **Year** | **Number of deaths** | **Women** | **Men** | **American Indian** | **Asian** | **Black African** | **White** | **Hispanic** | **Population** |
| --- | --- | --- | --- | --- | --- | --- | --- | --- | --- |
| 2018 | 3265 | 1552 | 1552 | - | 28 | 215 | 2998 | 113 | 327167434 |
| 2019 | 3638 | 1906 | 1732 | 21 | 36 | 240 | 3324 | 121 | 328239523 |
| 2020 | 4256 | 2273 | 1988 | 22 | 41 | 325 | 3854 | 148 | 329484123 |
| 2021 | 4619 | 2497 | 2127 | 21 | 41 | 290 | 4242 | 208 | 331893745 |
| 2022 | 4813 | 2564 | 2272 | 23 | 52 | 346 | 4390 | 154 | 333287557 |
| 2023 | 4510 | 2363 | 2164 | - | 61 | 316 | 4119 | 170 | 334914895 |

**Supplemental Table 2** UC and CD- related mortality, stratified by Place of death in general population in the United States, 2018 to 2023

| Year | Home | Inpatient facility | Nursing home/long term care | Hospice | Outpatient/ER facility | Dead on arrival | Others |
| --- | --- | --- | --- | --- | --- | --- | --- |
| 2018 | 997 | 1105 | 556 | 322 | 157 | 10 | 120 |
| 2019 | 1158 | 1216 | 593 | 368 | 181 | - | 116 |
| 2020 | 1548 | 1376 | 601 | 393 | 167 | - | 169 |
| 2021 | 1590 | 1644 | 630 | 367 | 189 | - | 201 |
| 2022 | 1714 | 1671 | 689 | 402 | 157 | - | 194 |
| 2023 | 1591 | 1529 | 681 | 411 | 160 | - | 160 |

**Supplemental Table 3** UC and CD- related mortality, stratified by annual trend in general population in the United States, 2018 to 2023

| **Year** | **Deaths** | **Population** | **Age Adjusted Rate** | **Age Adjusted Rate Lower 95% Confidence Interval** | **Age Adjusted Rate Upper 95% Confidence Interval** | **Age Adjusted Rate Standard Error** |
| --- | --- | --- | --- | --- | --- | --- |
| 2018 | 3267 | 327167434 | 8.269 | 7.981 | 8.558 | 0.147 |
| 2019 | 3638 | 328239523 | 9.13 | 8.828 | 9.432 | 0.154 |
| 2020 | 4261 | 329484123 | 10.483 | 10.163 | 10.803 | 0.163 |
| 2021 | 4624 | 331893745 | 11.583 | 11.243 | 11.923 | 0.174 |
| 2022 | 4836 | 333287557 | 11.632 | 11.298 | 11.966 | 0.17 |
| 2023 | 4527 | 334914895 | 10.761 | 10.442 | 11.08 | 0.163 |

**Supplemental Table 4** UC and CD- related mortality, stratified by sex trend in general population in the United States, 2018 to 2023

| **Sex** | **Year** | **Deaths** | **Population** | **Age Adjusted Rate** | **Age Adjusted Rate Lower 95% Confidence Interval** | | **Age Adjusted Rate Upper 95% Confidence Interval** | **Age Adjusted Rate Standard Error** |
| --- | --- | --- | --- | --- | --- | --- | --- | --- |
| Female | 2018 | 1715 | 166038755 | 7.778 | 7.401 | 8.155 | | 0.192 |
| Female | 2019 | 1906 | 166582199 | 8.605 | 8.209 | 9.001 | | 0.202 |
| Female | 2020 | 2273 | 167227921 | 9.99 | 9.569 | 10.411 | | 0.215 |
| Female | 2021 | 2497 | 167509003 | 11.29 | 10.837 | 11.743 | | 0.231 |
| Female | 2022 | 2564 | 168004004 | 11.116 | 10.675 | 11.557 | | 0.225 |
| Female | 2023 | 2363 | 169165495 | 10.165 | 9.747 | 10.583 | | 0.213 |
| Male | 2018 | 1552 | 161128679 |  | 8.418 | 9.318 | | 0.23 |
| Male | 2019 | 1732 | 161657324 | 9.789 | 9.319 | 10.259 | | 0.24 |
| Male | 2020 | 1988 | 162256202 | 11.001 | 10.508 | 11.494 | | 0.252 |
| Male | 2021 | 2127 | 164384742 | 11.914 | 11.395 | 12.433 | | 0.265 |
| Male | 2022 | 2272 | 165283553 | 12.28 | 11.764 | 12.796 | | 0.263 |
| Male | 2023 | 2164 | 165749400 | 11.436 | 10.943 | 11.928 | | 0.251 |

**Supplemental Table 5** UC and CD- related mortality, stratified by race in general population in the United States, 2018 to 2023

| **Race** | **Year** | **Deaths** | **Population** | **Age Adjusted Rate** | **Age Adjusted Rate Lower 95% Confidence Interval** | **Age Adjusted Rate Upper 95% Confidence Interval** | **Age Adjusted Rate Standard Error** |
| --- | --- | --- | --- | --- | --- | --- | --- |
| NH American Indian | 2019 | 21 | 4188092 | 6.263 | 3.771 | 9.781 | 1.423 |
| NH American Indian | 2020 | 22 | 4292990 | 6.049 | 3.745 | 9.247 | 1.334 |
| NH American Indian | 2021 | 21 | 4367540 | 5.386 | 3.29 | 8.319 | 1.218 |
| NH American Indian | 2022 | 23 | 4382234 | 5.52 | 3.46 | 8.358 | 1.181 |
| NH Asian | 2018 | 28 | 19330600 | 1.467 | 0.967 | 2.134 | 0.281 |
| NH Asian | 2019 | 36 | 19504862 | 1.9 | 1.324 | 2.643 | 0.32 |
| NH Asian | 2020 | 41 | 20012278 | 1.94 | 1.386 | 2.641 | 0.307 |
| NH Asian | 2021 | 41 | 20350483 | 1.894 | 1.353 | 2.579 | 0.3 |
| NH Asian | 2022 | 52 | 20953941 | 2.33 | 1.735 | 3.063 | 0.326 |
| NH Asian | 2023 | 61 | 21386914 | 2.708 | 2.066 | 3.486 | 0.349 |
| NH African American | 2018 | 215 | 43804319 | 5.167 | 4.458 | 5.875 | 0.361 |
| NH African American | 2019 | 240 | 44075086 | 5.752 | 5.006 | 6.497 | 0.38 |
| NH African American | 2020 | 325 | 44531112 | 7.368 | 6.547 | 8.19 | 0.419 |
| NH African American | 2021 | 290 | 45060857 | 6.742 | 5.944 | 7.54 | 0.407 |
| NH African American | 2022 | 346 | 45399743 | 7.758 | 6.919 | 8.597 | 0.428 |
| NH African American | 2023 | 316 | 45757433 | 6.897 | 6.116 | 7.678 | 0.398 |
| NH White | 2018 | 2998 | 250139096 | 9.135 | 8.801 | 9.469 | 0.17 |
| NH White | 2019 | 3324 | 250522190 | 10.05 | 9.701 | 10.4 | 0.178 |
| NH White | 2020 | 3854 | 250309724 | 11.515 | 11.143 | 11.887 | 0.19 |
| NH White | 2021 | 4242 | 251461611 | 12.88 | 12.484 | 13.277 | 0.202 |
| NH White | 2022 | 4390 | 251602174 | 12.879 | 12.489 | 13.268 | 0.199 |
| NH White | 2023 | 4119 | 252065704 | 11.948 | 11.575 | 12.321 | 0.19 |
| Hispanic | 2018 | 113 | 59871746 | 2.831 | 2.286 | 3.376 | 0.278 |
| Hispanic | 2019 | 121 | 60572237 | 2.808 | 2.288 | 3.328 | 0.265 |
| Hispanic | 2020 | 148 | 61312879 | 3.456 | 2.877 | 4.036 | 0.296 |
| Hispanic | 2021 | 208 | 62647044 | 4.744 | 4.07 | 5.418 | 0.344 |
| Hispanic | 2022 | 154 | 63664346 | 3.291 | 2.751 | 3.832 | 0.276 |
| Hispanic | 2023 | 170 | 65219145 | 3.651 | 3.081 | 4.221 | 0.291 |

**Supplemental Table 6** UC and CD- related mortality, stratified by state in general population in the United States, 2018 to 2023

| **State** | **Deaths** | **Population** | **Age Adjusted Rate** | | **Age Adjusted Rate Lower 95% Confidence Interval** | **Age Adjusted Rate Upper 95% Confidence Interval** | **Age Adjusted Rate Standard Error** |
| --- | --- | --- | --- | --- | --- | --- | --- |
| Alabama | 241 | 29935229 | 6.617 | 5.756 | | 7.477 | 0.439 |
| Alaska | 50 | 4399803 | 11.587 | 8.451 | | 15.504 | 1.724 |
| Arizona | 511 | 43938621 | 9.083 | 8.276 | | 9.89 | 0.412 |
| Arkansas | 216 | 18201411 | 9.462 | 8.172 | | 10.753 | 0.659 |
| California | 2284 | 235669717 | 8.467 | 8.115 | | 8.818 | 0.179 |
| Colorado | 594 | 34791624 | 15.633 | 14.355 | | 16.911 | 0.652 |
| Connecticut | 225 | 21543936 | 7.789 | 6.753 | | 8.824 | 0.528 |
| Delaware | 66 | 5981414 | 7.972 | 6.139 | | 10.18 | 0.996 |
| District of Columbia | 28 | 4141845 | 6.561 | 4.324 | | 9.546 | 1.26 |
| Florida | 1770 | 131147051 | 9.182 | 8.738 | | 9.625 | 0.226 |
| Georgia | 568 | 64588584 | 8.114 | 7.434 | | 8.794 | 0.347 |
| Hawaii | 41 | 8560256 | 3.416 | 2.417 | | 4.689 | 0.552 |
| Idaho | 237 | 11172868 | 18.061 | 15.72 | | 20.401 | 1.194 |
| Illinois | 676 | 75803621 | 7.268 | 6.71 | | 7.826 | 0.285 |
| Indiana | 601 | 40680271 | 12.312 | 11.309 | | 13.315 | 0.512 |
| Iowa | 307 | 19075376 | 12.125 | 10.742 | | 13.509 | 0.706 |
| Kansas | 196 | 17550902 | 9.305 | 7.971 | | 10.638 | 0.68 |
| Kentucky | 439 | 26961184 | 13.307 | 12.036 | | 14.578 | 0.648 |
| Louisiana | 245 | 27742127 | 7.52 | 6.554 | | 8.486 | 0.493 |
| Maine | 186 | 8186066 | 14.674 | 12.49 | | 16.858 | 1.114 |
| Maryland | 642 | 36654242 | 14.321 | 13.196 | | 15.446 | 0.574 |
| Massachusetts | 537 | 41656322 | 9.926 | 9.071 | | 10.781 | 0.436 |
| Michigan | 893 | 60071512 | 11.473 | 10.7 | | 12.247 | 0.395 |
| Minnesota | 787 | 34070642 | 18.485 | 17.172 | | 19.797 | 0.67 |
| Mississippi | 193 | 17759177 | 9.342 | 7.985 | | 10.698 | 0.692 |
| Missouri | 398 | 36957728 | 8.436 | 7.588 | | 9.283 | 0.432 |
| Montana | 118 | 6571610 | 13.149 | 10.707 | | 15.591 | 1.246 |
| Nebraska | 252 | 11711222 | 18.026 | 15.756 | | 20.296 | 1.158 |
| Nevada | 216 | 18768746 | 9.792 | 8.459 | | 11.125 | 0.68 |
| New Hampshire | 143 | 8268721 | 11.985 | 9.968 | | 14.002 | 1.029 |
| New Jersey | 558 | 54492751 | 8.081 | 7.398 | | 8.764 | 0.349 |
| New Mexico | 120 | 12642168 | 7.304 | 5.966 | | 8.643 | 0.683 |
| New York | 1223 | 117416826 | 8.085 | 7.623 | | 8.548 | 0.236 |
| North Carolina | 727 | 63558153 | 9.43 | 8.731 | | 10.129 | 0.357 |
| North Dakota | 71 | 4625583 | 13.458 | 10.408 | | 17.122 | 1.651 |
| Ohio | 1055 | 70393769 | 11.617 | 10.899 | | 12.334 | 0.366 |
| Oklahoma | 481 | 23941096 | 17.04 | 15.486 | | 18.594 | 0.793 |
| Oregon | 691 | 25369607 | 21.078 | 19.481 | | 22.676 | 0.815 |
| Pennsylvania | 1129 | 77290050 | 10.623 | 9.987 | | 11.258 | 0.324 |
| Rhode Island | 130 | 6459107 | 15.079 | 12.428 | | 17.731 | 1.353 |
| South Carolina | 438 | 31297775 | 11.138 | 10.063 | | 12.213 | 0.549 |
| South Dakota | 99 | 5384129 | 15.318 | 12.349 | | 18.786 | 1.593 |
| Tennessee | 578 | 41639064 | 11.564 | 10.6 | | 12.528 | 0.492 |
| Texas | 1469 | 177119299 | 8.377 | 7.943 | | 8.812 | 0.222 |
| Utah | 270 | 19753451 | 15.793 | 13.885 | | 17.701 | 0.973 |
| Vermont | 109 | 3813733 | 20.186 | 16.258 | | 24.114 | 2.004 |
| Virginia | 625 | 51685358 | 10.058 | 9.257 | | 10.859 | 0.409 |
| Washington | 796 | 46181454 | 14.686 | 13.649 | | 15.724 | 0.529 |
| West Virginia | 204 | 10710952 | 14.127 | 12.101 | | 16.153 | 1.034 |
| Wisconsin | 644 | 35168059 | 14.137 | 13.021 | | 15.253 | 0.569 |
| Wyoming | 76 | 3483065 | 17.554 | 13.735 | | 22.106 | 2.075 |

**Supplemental Table 7** UC and CD- related mortality, stratified by census region in general population in the United States, 2018 to 2023

| **Census Region** | **Year** | **Deaths** | **Population** | **Age Adjusted Rate** | **Age Adjusted Rate Lower 95% Confidence Interval** | **Age Adjusted Rate Upper 95% Confidence Interval** | **Age Adjusted Rate Standard Error** |
| --- | --- | --- | --- | --- | --- | --- | --- |
| Census Region 1: Northeast | 2018 | 591 | 56111079 | 8.205 | 7.527 | 8.884 | 0.346 |
| Census Region 1: Northeast | 2019 | 640 | 55982803 | 8.766 | 8.071 | 9.462 | 0.355 |
| Census Region 1: Northeast | 2020 | 747 | 55849869 | 10.111 | 9.367 | 10.855 | 0.379 |
| Census Region 1: Northeast | 2021 | 798 | 57159838 | 10.713 | 9.953 | 11.473 | 0.388 |
| Census Region 1: Northeast | 2022 | 754 | 57040406 | 9.765 | 9.054 | 10.477 | 0.363 |
| Census Region 1: Northeast | 2023 | 710 | 56983517 | 9.127 | 8.443 | 9.811 | 0.349 |
| Census Region 2: Midwest | 2018 | 760 | 68308744 | 8.901 | 8.255 | 9.547 | 0.33 |
| Census Region 2: Midwest | 2019 | 824 | 68329004 | 9.616 | 8.945 | 10.286 | 0.342 |
| Census Region 2: Midwest | 2020 | 1068 | 68316744 | 12.26 | 11.51 | 13.011 | 0.383 |
| Census Region 2: Midwest | 2021 | 1105 | 68841444 | 13.113 | 12.323 | 13.903 | 0.403 |
| Census Region 2: Midwest | 2022 | 1146 | 68787595 | 13.05 | 12.277 | 13.823 | 0.394 |
| Census Region 2: Midwest | 2023 | 1076 | 68909283 | 12.244 | 11.497 | 12.992 | 0.381 |
| Census Region 3: South | 2018 | 1117 | 124753948 | 7.537 | 7.088 | 7.987 | 0.229 |
| Census Region 3: South | 2019 | 1284 | 125580448 | 8.551 | 8.076 | 9.027 | 0.243 |
| Census Region 3: South | 2020 | 1454 | 126662754 | 9.534 | 9.035 | 10.034 | 0.255 |
| Census Region 3: South | 2021 | 1632 | 127225329 | 10.831 | 10.295 | 11.367 | 0.274 |
| Census Region 3: South | 2022 | 1778 | 128716192 | 11.384 | 10.846 | 11.922 | 0.275 |
| Census Region 3: South | 2023 | 1665 | 130125290 | 10.441 | 9.931 | 10.951 | 0.26 |
| Census Region 4: West | 2018 | 799 | 77993663 | 8.953 | 8.324 | 9.583 | 0.321 |
| Census Region 4: West | 2019 | 890 | 78347268 | 9.946 | 9.283 | 10.61 | 0.338 |
| Census Region 4: West | 2020 | 992 | 78654756 | 10.747 | 10.069 | 11.426 | 0.346 |
| Census Region 4: West | 2021 | 1089 | 78667134 | 12.094 | 11.365 | 12.824 | 0.372 |
| Census Region 4: West | 2022 | 1158 | 78743364 | 12.271 | 11.555 | 12.986 | 0.365 |
| Census Region 4: West | 2023 | 1076 | 78896805 | 11.312 | 10.628 | 11.997 | 0.349 |
